# Recognizing the diagnostic challenges of mammary Paget’s disease: a case report

**DOI:** 10.1097/JW9.0000000000000259

**Published:** 2026-02-25

**Authors:** Hiba A. Mohammed, Farrukh Azmi, Leanne Avery, Valerie Harvey

**Affiliations:** a Department of Medicine, Henry Ford Health, Jackson, Michigan; b Department of Dermatopathology, LabCorp, Burlington, North Carolina; c Department of Dermatology, Hampton Roads Center for Dermatology, Newport News, Virginia; d Department of Dermatology, Edward Via College of Osteopathic Medicine, Blacksburg, Virginia

**Keywords:** breast cancer, diagnostic challenges, ductal carcinoma in situ, mammary Paget’s disease, Paget’s disease

**What is known about this subject in regard to women and their families?**

- Mammary Paget’s disease predominantly affects women over 50 and is strongly associated with underlying breast carcinoma.

**What is new from this article as messages for women and their families?**

- This article highlights the diagnostic challenges associated with mammary Paget’s disease and emphasizes the importance of histopathologic evaluation for early detection and management in women.

## Introduction

Mammary Paget disease (MPD) is an uncommon intraepithelial malignancy affecting the nipple and areola and is often associated with underlying breast carcinoma. Malignant cells invade the nipple and adjacent skin, leading to the formation of an eczematous lesion.^1^ We present a case of MPD with microinvasive ductal adenocarcinoma in a 56-year-old woman.

## Case report

A 56-year-old woman presented with a 6-month history of a painful, bleeding left nipple lesion. Her medical history is pertinent for a left lumpectomy for a benign breast mass.

**Fig. 1.**
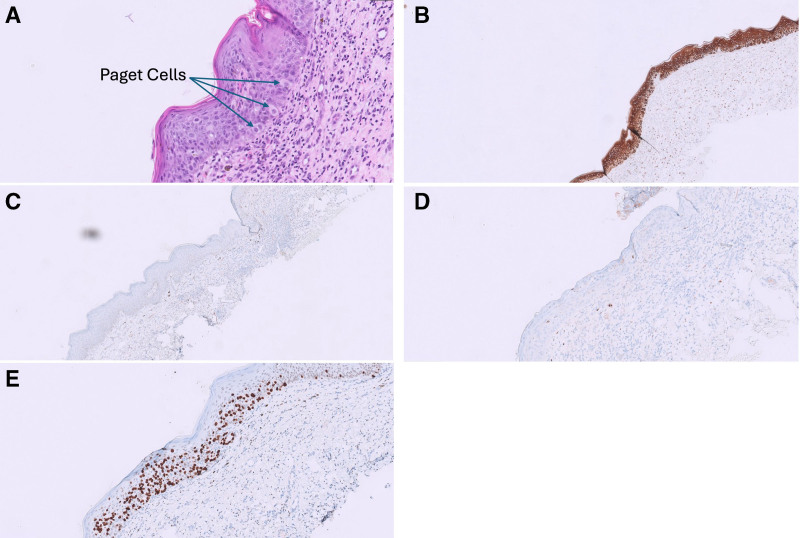
Histological and immunohistochemical staining of MPD: (A) H&E stain showing intraepidermal proliferation of atypical epithelial cells (Paget cells) and presence of inflammatory cells in dermis. (B) Pankeratin positivity. (C) CK-7 negative. (D) CK-20 negative. (E) GATA3 positivity. H&E, hematoxylin and eosin; MPD, mammary Paget’s disease.

Examination revealed a 0.8 cm excoriated plaque involving the left areola without associated lymphadenopathy. Biopsy of the left areola showed intraepidermal proliferation of atypical epithelial cells. Immunohistochemistry revealed positive Pan-Keratin, GATA3, and estrogen receptor (ER), but negative S100, Melan-A, CK7, CK20, and progesterone receptor (PR) (Fig. 1). Histopathology results were consistent with Paget’s disease.

Diagnostic mammography, ultrasound, and magnetic resonance imaging (MRI) revealed no underlying malignancy. Patient subsequently underwent a left central mastectomy and sentinel lymph node biopsy. Pathology revealed high-grade microinvasive ductal carcinoma in situ with Paget’s of the left nipple, measuring 0.77 mm, with a negative sentinel lymph node and clear margins. Hormone receptor studies demonstrated ER positivity (>95% moderate nuclear staining), PR positivity (90% moderate-strong nuclear staining), and Her-2/neu negative. Patient followed up with oncology, has initiated adjuvant radiation therapy, and will start tamoxifen upon completion of radiation.

## Discussion

MPD is a rare manifestation of breast cancer, affecting 1 to 3% of patients.^1^ Most cases (93–100%) are associated with underlying ductal carcinoma in situ or invasive adenocarcinoma.^2^ Clinically, MPD presents as an eczematous plaque of the nipple and areolar skin with secondary crusting, scaling, or ulceration.^2^ Misdiagnosis is common, as it can mimic nipple eczema, Bowen disease, psoriasis, and cutaneous melanoma.^3^

Diagnosis requires histopathology, revealing malignant epidermal Paget cells, accompanied by dermal inflammation or telangiectasias.^2^ Immunohistochemistry is important in differential diagnosis and subtype classification. MPD typically expresses CK7 (>90%), HER2 (80–100%), CAM5.2 (70–100%), CEA (71%), and variable ER (10–40%) and PR (28.6%) expression, but lacks CK10, CK14, and CK20.^2,4^ In contrast to typical findings, our patient’s biopsy showed positive Pan-Keratin, GATA3, and ER but negative S100, Melan-A, CK7, CK20, and PR. This discrepancy aligns with recent studies highlighting GATA3 as a reliable MPD marker, positive in 95% of cases, even when CK7 is negative.^4^ Poorly differentiated breast cancer often shows HER2/neu overexpression and lacks ER/PR expression.^5^ S100, Melan-A, and HMB-45 negativity aid in excluding melanoma.^1^

Imaging is essential in detecting carcinoma and guiding management. Bilateral mammography is recommended but has reduced sensitivity in cases without a palpable mass.^3^ Ultrasound may be helpful when mammography is negative, but it may not always detect underlying cancer.^3^ MRI has greater sensitivity and is suggested for patients with negative mammography, but a small risk of false negatives remains.^1^ Our patient’s mammography, ultrasound, and MRI all showed no evidence of underlying malignancy.

MPD poses diagnostic challenges due to its variable immunohistochemical profile, inconsistent imaging findings, and diverse presentations. Our case highlights the importance of considering MPD in persistent nipple dermatoses and the critical role of histopathologic evaluation for early detection and management.

## Conflicts of interest

None.

## Funding

None.

## Study approval

N/A.

## Author contributions

HAM: Drafted the manuscript. FA: Provided the histopathology report and contributed the data in Figure 1. LA: Conducted the patient interviews. VH: Provided clinical expertise and guidance and contributed to manuscript revision. All authors reviewed the final manuscript. The authors declare no conflicts of interest.

## Patient consent

Informed, written consent was received from all patients for whom photographs are present in the manuscript.
